# Field Based Assessment of *Capsicum annuum* Performance with Inoculation of Rhizobacterial Consortia

**DOI:** 10.3390/microorganisms7030089

**Published:** 2019-03-21

**Authors:** Manoj Kaushal, Priyanka Mandyal, Rajesh Kaushal

**Affiliations:** 1 International Institute of Tropical Agriculture, Mikocheni B, Dar es Salaam 34441, Tanzania; 2 Department of Basic Sciences, Dr. Y.S. Parmar University of Horticulture and Forestry, Nauni, Solan, HP 173230, India; priyanka.1631@rediffmail.com (P.M.); drrkaushal@rediffmail.com (R.K.)

**Keywords:** bell pepper, anthracnose, damping off, disease suppression, principal component analysis

## Abstract

Plant growth promoting rhizobacteria (PGPR) are associated with plant roots and augment plant productivity and immunity by reducing fertilizer application rates and nutrient runoff. Studies were conducted to evaluate bell pepper transplants amended with formulation of consortium of two indigenous PGPR isolates (*Bacillus subtilis* and *Bacillus pumilus*) in terms of increase in yield and disease resistance under field conditions. Transplants were planted into plots treated by NPK (nitrogen, phosphorus and potassium), fungicides, soil solarization, MeBr fumigation, PGPR and untreated soil. Treatments were assessed for incidence of soil-borne phytopathogens viz. *Phytophthora capsici* and *Colletotrichum* sp. Highly significant increases in bell pepper transplant growth occurred in response to formulations of PGPR isolates. Transplant vigor and survival in the field were also improved by PGPR treatments. Consortium of *Bacillus subtilis* and *Bacillus pumilus* reduced disease incidence of damping off by 1.81% and anthracnose by 1.75%. Numbers of colony forming units of *Phytophthora capsici* and *Colletotrichum* sp. were significantly higher in all plots than those treated with PGPR consortium. Incidence of seed rot and seedling blight on bell pepper was significantly lower in PGPR-treated plots and highest in untreated plots. Total fruit yield of bell pepper increased by 379.36% with PGPR consortium (*Bacillus subtilis* and *Bacillus pumilus*).

## 1. Introduction

*Capsicum annuum* L. commonly called bell or sweet pepper is among the most popular vegetable crop cultivated worldwide. It has been widely accepted as a valuable nutritious food due to presence of vitamins (A, C, E and K1) and antioxidants. Its color, aroma, flavor and crunchy texture are major attributes for its wide acceptance in various culinary dishes around the world [1]. In the 19^th^ century, the British introduced bell pepper to the Shimla hills, and since then it is generally been referred to as “Shimla Mirch” in India and is an important vegetable cultivated chiefly in the mid-hills of Himachal Pradesh. In India, other commercial primarily capsicum producing regions are Tamil Nadu, Karnataka, Uttar Pradesh, and the Deccan Plateau. However, due to lesser production rates and small areas under cultivation, there is not enough for adequate supply in comparison to US, Holland, France and other capsicum producing countries around the world. Moreover, damping off and anthracnose caused by *Phytophthora capsici* and *Colletotrichum* spp. respectively, are major devastative and destructive diseases which drastically reduces potential crop yields [2]. Damping off is a soil-borne fungal disease of seedlings caused by *Phytopthora* which can be the bane of the amateur seed and occurs on the seeding table when young plants are just beginning to grow. During pre-emergence damping off, the seeds may rot before germinating or seedlings may die prior to emergence. If it is post-emergence damping off, the young plant seedlings develop a rot at the crown. Later, the tissue becomes soft resulting in wilting of the plant and its fall over its base [3]. Anthracnose is another common disease and is caused by the fungus *Colletotrichum* also called ripe-fruit rot. The disease reduces the productivity of the ripened fruits of sweet pepper and turns them into rotted waste in just a few days [2]. Symptoms appear mostly as circular, sunken lesions or spots that appear on the fruit, with black margins and covered with a pinkish mass of fungal spores. With disease progression, the spots spread, forming concentric lesions with dark fructifications representing the fungal fruiting structures called acervuli [3]. Various chemical fungicides provide good results in coping with these soil-borne diseases and fungal pathogens but excessive use can cause various environmental hazards and carcinogenic and mutagenic effects in living beings [4]. Hence, plant protection is the need of the hour to maintain sustainable crop yields. Alternatively, to combat production loss due to harmful diseases, it is essential to consider the need for biological control agents (BCAs). BCAs are an alternative to chemical fertilizers and are a safe and efficient tool against phytopathogens [5].

Plant growth promoting rhizobacteria (PGPR) such as *Bacillus* and *Pseudomonas* are very well-known for biological control against various soil-borne phytopathogens. They colonize plant roots and provide direct and indirect effects. Various mechanisms of PGPR include increased nitrogen uptake, synthesis of indoleacetic acid (IAA), phosphate solubilization, suppression of soil-borne pathogens by producing siderophores, etc., which enhance plant growth and yield [6,7]. Plant disease resistance is again supposed to be a dynamic and multifactorial process. It is assumed that *Bacillus* spp. suppress soil borne fungal pathogens by a mechanism called induced systemic resistance [8]. Positive impacts of PGPR on the initial growth of cauliflower and other vegetable crops and its ability to attenuate various soil-borne diseases has been described previously [9]. Many researchers found that among selected PGPR isolates, four significantly decreased gray leaf spot disease severity with PGPR *Brevibacterium iodinum* (KUDC1716) providing the highest disease suppression in bell pepper (*Capsicum annuum*) [10]. In another greenhouse study, lower degrees of root rot and taller plants were found by the application of *Burkholderia cepacia* (BRB21) in pepper [11]. However, few studies have been reported for suppression of diseases caused by *Phytophthora capsici* and *Colletotrichum* spp. in bell pepper and are restricted to net/glasshouse studies. In this study, we systematically investigated the biocontrol efficacies of a consortium of two already isolated PGPR strains (*Bacillus pumilus*, YSPMK11 and *Bacillus subtilis*, MK_5_) against damping off and anthracnose diseases on field grown peppers. Hence, the present investigation was planned and executed with two objectives. First, to evaluate the growth promotion efficacies by a consortium (*Bacillus pumilus*, YSPMK11 and *Bacillus subtilis*, MK_5_) on bell pepper. Second is to test the efficacy of a consortium in controlling damping off and anthracnose diseases in bell pepper under field conditions.

## 2. Materials and Methods

### 2.1. Procurement and Mass Culturing of Microbial Strains

The isolates of PGPR’s (*Bacillus pumilus* and *Bacillus subtilis*) were procured from Soil Microbiology Lab of Dept. of Soil Science and Water Management, Dr. Y S Parmar, University of Horticulture and Forestry, Solan (Himachal Pradesh), India. Both strains are indigenous and isolated from our previous study [9,12] and were multiplied on nutrient broth medium (MM 244, HiMedia Laboratories). The nutrient broth medium containing 0.5% of peptone and sodium chloride and 0.3% of beef extract were suspended in 1 L of distilled water and autoclaved at 15 lbs of pressure (121 °C) for 15 min. For making the stock solution, each bacterial strain (*Bacillus pumilus* and *Bacillus subtilis*) was grown for 24 h at 30 °C with rotation at 120 rpm until reaching the exponential phase. After centrifugation, the cultures were again resuspended in sterile water and adjusted to a final concentration of ~10^9^ CFU/mL for use as inoculum.

### 2.2. Characterization of PGPR Isolates

Both the isolates (*Bacillus pumilus* and *Bacillus subtilis*) were characterized for their in vitro multifarious plant growth promoting traits (phosphate solubilization, IAA and siderophore production). Cultures were also tested for their antagonistic activities against *Phytophthora capsici* and *Colletotrichum* spp. Phosphate solubilization potential was detected utilizing PVK (Pikovskaya’s) broth. Quantitative estimation of water extractable free inorganic P (Pi) was then carried out. IAA and siderophore production were determined [9,12]. IAA production was analysed using a colorimetric method. The isolates were incubated in yeast malt dextrose broth at 28 °C for 96 h followed by centrifugation. Thereafter, 1 mL of supernatant was mixed with 2 mL of Salkowski reagent and optical density was recorded at 530 nm. For siderophore production overnight cultures of both isolates were inoculated onto chrome azurol S (CAS) agar plates and incubated at 28 °C for 72 h. Isolates with an orange halo zone were considered positive for siderophore production and the diameter of halo zone was measured.

Seed of cv. California Wonder obtained from the Seed Technology and Production Centre of Dr. Y S Parmar University of Horticulture and Forestry. Seeds were surface sterilized in 0.2% of HgCl_2_ for 3 min and washed with sterilized distilled water several times. Seed emergence was also analysed in a growth chamber using consortia of bacterial strains and compared with an uninoculated control. Nitrogen fixing capacities of both the indigenous strains were quantified indirectly by an acetylene reduction assay (ARA) [13].

### 2.3. Field Experiments to Evaluate Performance of PGPR’s Consortia

Field experiments were conducted during 2010–2011 at the Research Farm in Solan (Himachal Pradesh), replicated thrice. The elevation is 1523 m above mean sea level and the site has moderate-to-heavy monsoon rains. Seedlings, approximately six-weeks-old, were transplanted into beds with plants spaced at 30 cm intervals within the row. Each plot consisted of two 7.5 m rows, spaced 0.8 m apart with 25 plants per row. Main plots consisted of seven soil treatments arranged in a randomized complete block design with five replications. Main plot treatments were: (1) Control—PM1, (2) Benomyl (fungicide)—PM3, (3) Captan (fungicide)—PM4, (4) Recommended dose of NPK—PM6, (5) Soil fumigation with MeBr (425 kg/ha broadcast 98:2 MeBr:chloropicrin)—PM7, (6) PGPR’s consortium (*Bacillus pumilus* and *Bacillus subtilis*)—PM9, (7) Soil solarisation—PM10. Doses of fungicides and NPK (5-10-10) used in these treatments were as commonly used by local bell pepper growers. The plants were watered by a micro-irrigation system [14] to maintain a 50% moisture content in soil. Rhizobacterial cultures were incubated for 72 h and then diluted to a final concentration of 9 × 10^8^ CFU/mL in 10% nutrient broth. Seeds were inoculated with rhizobacterial consortia by soaking in a Petri dish before nursery sowing for 30 min at 1 mL/seed. The liquid cultures of rhizobacterial consortia were also used for inoculations as soil drench at 50% bloom stage at 10 mL/plant [9]. Plants were harvested after 90 days and recorded for height and root length recorded. Dry weight of shoots and roots was determined after drying in an oven at 65 ± 5 °C for 72 h. Numbers of fruits/plant were counted, and average fruit yield/plant was calculated. The incidence of damping off and anthracnose were assessed in the field based on the percentage of stems with disease symptoms. Ten randomly selected bell pepper plants with disease symptoms from each treatment plots were chosen. Disease severity and control efficacy were calculated as follows: Disease severity = [∑ (The number of diseased plants in this index × Disease index)/(Total number of plants investigated × The highest disease index)] × 100%. Control efficacy = [(Disease severity of control–Disease severity of treated group)/Disease severity of control] × 100%.

### 2.4. Statistical Analysis

Statistical analysis was done using the statistical package SAS version 9.3 (SAS Institute Inc., Cary, NC, USA). Data were analyzed by analysis of variance (ANOVA) and compared using least significant difference at 5% level of significance (*α* = 0.05). Principal component analysis was performed to compare the means of the treatments with yield parameters and diseases suppression.

## 3. Results and Discussion

### 3.1. Characterization of PGPR Isolates

**Figure 1:**
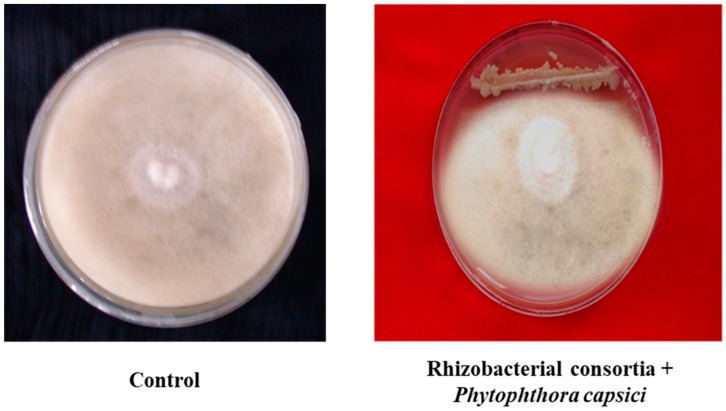

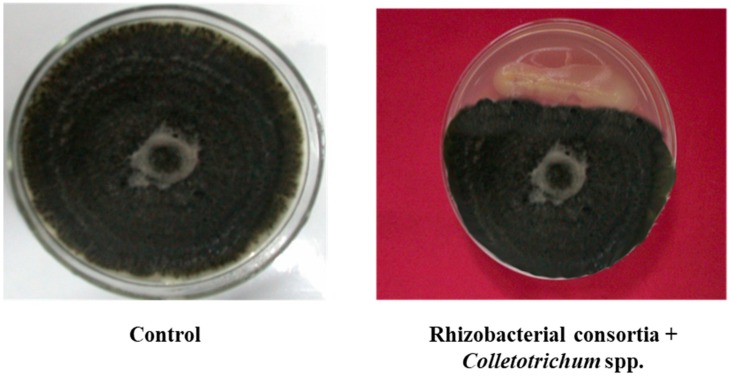
Growth inhibition of mycelia *Phytophthora capsici* and *Colletotrichum* sp. by rhizobacterial consortium.

**Figure 2:**
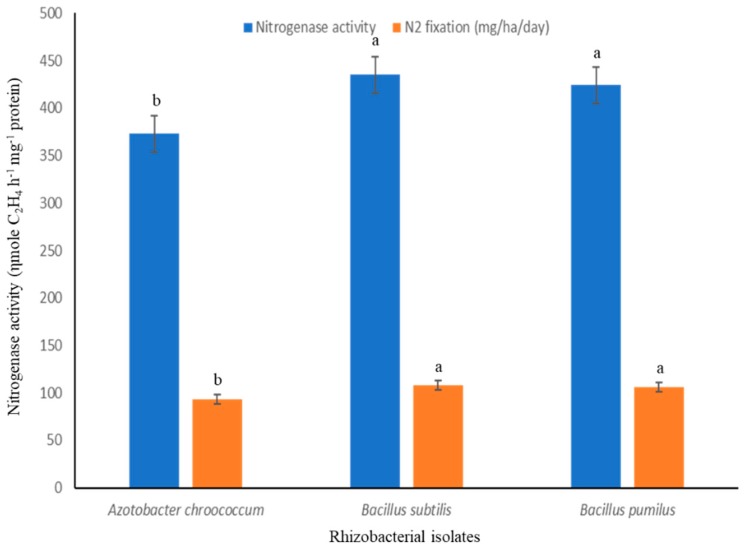
Nitrogenase activity of the PGPR’s isolates over reference strain. Different letters mean significant differences among categories (*p* < 0.05). Error bars indicate ± SD.

Both the isolates (*Bacillus pumilus* and *Bacillus subtilis*) were found positive for phosphate-solubilizing assays, IAA, and siderophore production. An increase in phosphorous-solubilization occurred between 48–72 h of incubation. The maximum amount of P-solubilization (287.50 µg/mL) with *Bacillus pumilus* and (279.35 µg/mL) by *Bacillus subtilis*, was at 96 h of incubation. Indole acetic acid production by *Bacillus pumilus* and *Bacillus subtilis* was found to increase with increasing concentration of l-tryptophan supplementation from 0 to 500 µg/mL. The highest level, 78.7 µg/mL, was produced by isolate *Bacillus pumilus* followed by *Bacillus subtilis* (72.0 µg/mL) when the media was supplemented with 500 µg/mL of l-tryptophan. It was investigated that IAA is the most important plant hormone produced by rhizobacterial strains which is directly involved in plant growth promotion [15]. The production of siderophores, low molecular weight iron chelating compounds, was detected in both isolates, conferring them a competitive merit to biocontrol agents. This contributed to disease suppression due to the limited supply of essential trace elements in the soil [16]. The production of siderophores was confirmed by 8 mm to 10 mm yellowish orange zone produced by *Bacillus pumilus* and *Bacillus subtilis*. The potential of beneficial rhizobacteria with multifarious plant growth promoting attributes to improve plant growth, including root growth, shoot growth, fresh weight, dry weight, branches/tillers, root biomass, shoot biomass, flowering, pods/grains etc. was investigated in various studies [17,18]. In in vitro tests, consortium of both the isolates showed varied levels of inhibition in mycelia growth of *Phytophthora capsici* and *Colletotrichum* sp. (Figure 1). The surface of the uninoculated control plates were completely covered by the phytopathogens, displaying no inhibition. But the inoculated plates with consortium of both native bacterial strains showed 85% and 88.50% inhibition of mycelia growth for *Phytophthora capsici* and *Colletotrichum* sp., respectively. Data indicated that rhizobacterial consortium inhibits *Phytophthora capsici* and *Colletotrichum* sp. by means of antibiosis [19,20] as the fungal antagonists *B. subtilis* and *B. pumilus* have been shown to be effective biocontrol agents in prior studies. The comparison of nitrogenase activity of both the efficient nitrogen-fixing isolates (*Bacillus pumilus* and *Bacillus subtilis*) over reference strain (*Azotobacter chroococcum*) procured from the Institute of Microbial Technology, Chandigarh is shown in (Figure 2). Both the rhizobacterial strains showed higher nitrogenase activity, i.e., 435.26 ηmole C_2_H_4_ h^−1^ mg^−1^ protein (108 mg of N_2_ fixed/ha/day) by *Bacillus subtilis* and 424.25 ηmole C_2_H_4_ h^−1^ mg^−1^ protein (106 mg of N_2_ fixed/ha/day) by *Bacillus pumilus* as compared to standard strain of *Azotobacter chroococcum*, i.e., 372.85 ηmole C_2_H_4_ h^−1^ mg^−1^ protein (93.0 mg of N_2_ fixed/ha/day).

### 3.2. Field Experiments to Evaluate Performance of PGPR’s Consortia

**Figure 3:**
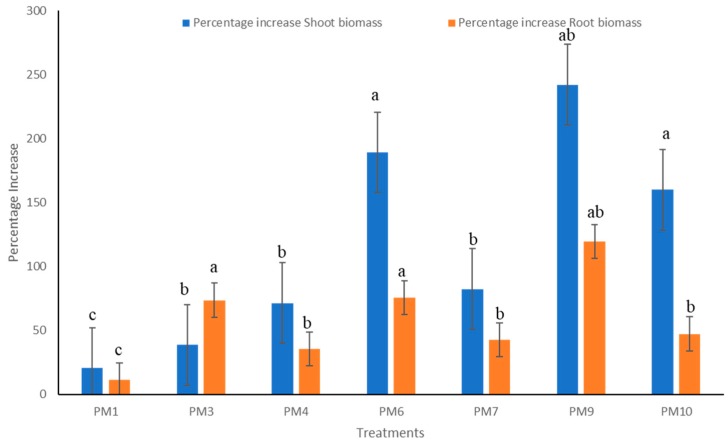
Impact of PGPR’s consortium on shoot and root biomass under field conditions. Different letters mean significant differences among categories (*p* < 0.05). Error bars indicate ± SD. PM1—Control, PM3—Benomyl, PM4—Captan, PM6—Recommended dose of NPK, PM7—Soil fumigation with MeBr (425 kg/ha broadcast 98:2 MeBr:chloropicrin), PM9—PGPR’s consortium (*Bacillus pumilus* and *Bacillus subtilis*), PM10—Soil solarization.

**Figure 4:**
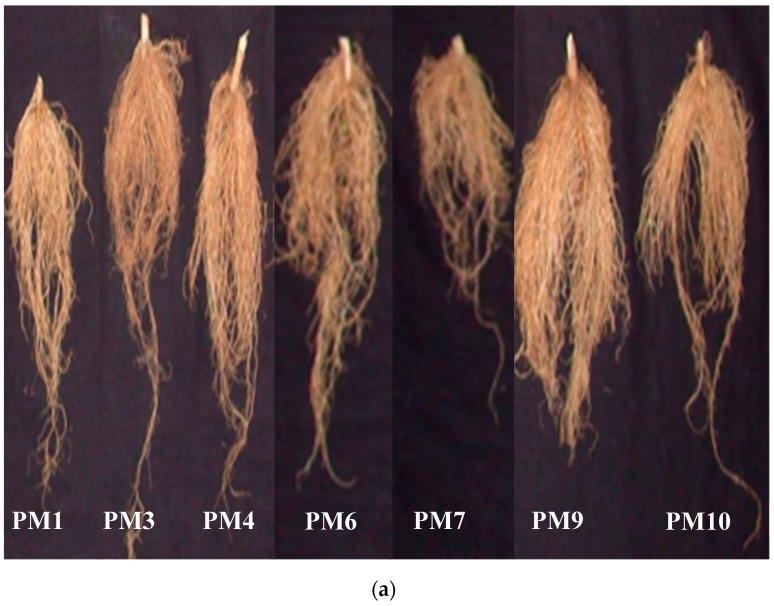

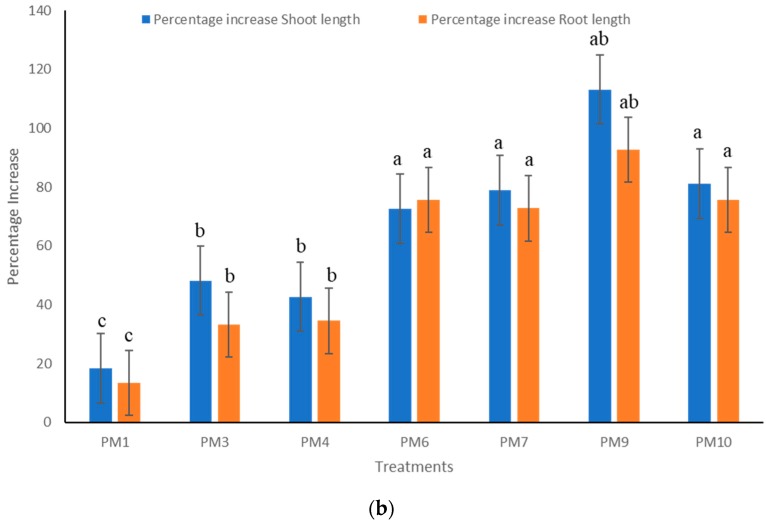
Impact of PGPR’s consortium on (**a**) root growth and (**b**) shoot and root length under field conditions. Different letters mean significant differences among categories (*p* < 0.05). Error bars indicate ± SD. PM1—Control, PM3—Benomyl, PM4—Captan, PM6—Recommended dose of NPK, PM7—Soil fumigation with MeBr (425 kg/ha broadcast 98:2 MeBr:chloropicrin), PM9—PGPR’s consortium (*Bacillus pumilus* and *Bacillus subtilis*), PM10—Soil solarization.

**Figure 5:**
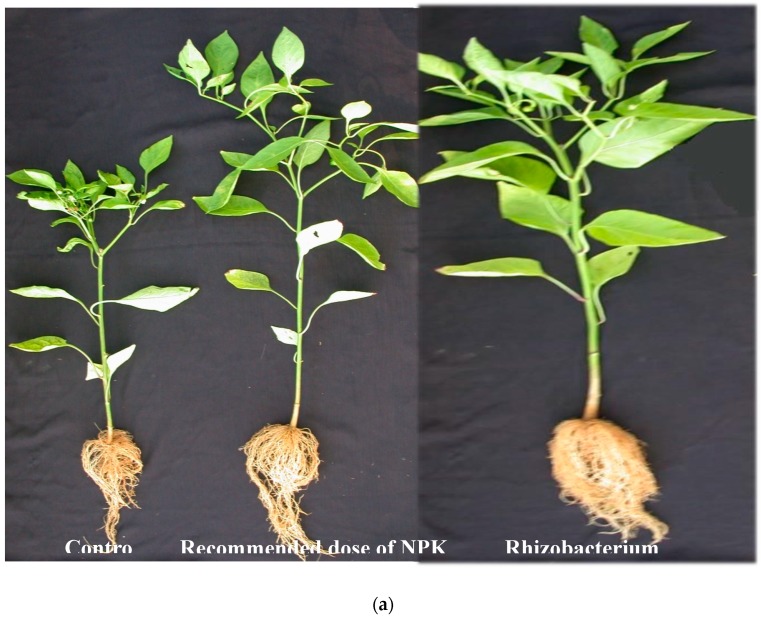

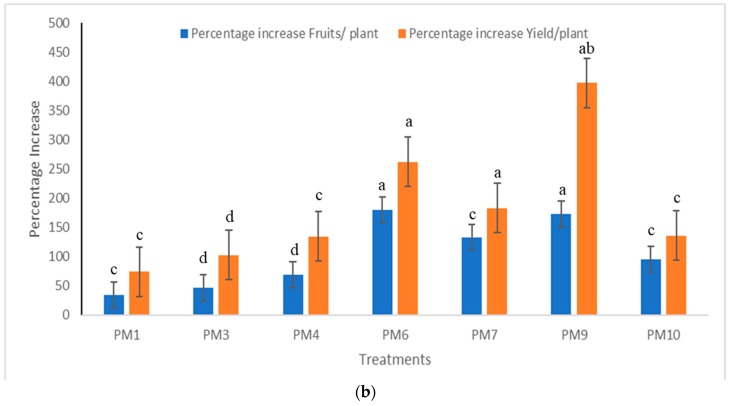
Impact of PGPR’s consortium on (**a**) growth of plants (**b**) growth and yield of capsicum under field conditions. Different letters mean significant differences among categories (*p* < 0.05). Error bars indicate ± SD. PM1—Control, PM3—Benomyl, PM4—Captan, PM6—Recommended dose of NPK, PM7—Soil fumigation with MeBr (425 kg/ha broadcast 98:2 MeBr:chloropicrin), PM9—PGPR’s consortium (*Bacillus pumilus* and *Bacillus subtilis*), PM10—Soil solarization.

Rhizobacterial consortium significantly increased plant growth for almost all parameters measured during every replication of the study. Plants treated with PM9 produced a maximum of 242.1% increase in shoot biomass and 119.6% increase in root biomass over uninoculated controls (Figure 3). Plots inoculated with PM9 also increased shoot length (113.2%) and root length (92.8%) over inoculated control which was maximum among all the other applied treatments (Figure 4). Increase in root growth was due to the plant growth stimulating phytohormones produced by rhizobacterial consortium in the vicinity of roots. Rhizobacterial consortium also stimulated the adventitious root development and thus increased the density and length of roots including the number of root hairs [21]. This also increased the root surface area with improved potential for water and nutrient uptake by the host plant and led to enhanced shoot growth [22]. In addition to highly significant growth promotion in pepper, transplant vigor and survival in the field improved with PGPR’s formulations (PM9). This is best illustrated by the significant enhancement of pepper transplant survival at 15 DAP (days after planting). Yield of pepper was also uplifted by rhizobacterial consortium (PM9) by 397.36% over uninoculated controls. The most dramatic effect was observed with PM6, which resulted in increases in fruits/plant by 173.17% (maximum) however, it was significantly at par with the PM9 (172.7%) treated plot (Figure 5). This enhancement could be due to the growth of pepper plants in PM6 occurring because of the applied recommended dose of NPK fertilizers (Figure 5). Production of IAA plant hormones by both the rhizobacterial strains are the major impacting factor responsible for direct stimulation of shoot and root growth as well as improvement of host health [23].

The consortium of isolates was further evaluated for their efficacy to suppress damping off and anthracnose of pepper under field conditions. For comparative analysis, a set of treatments (fertilizers, locally used fungicides, soil solarization and fumigation) were maintained in different plots. It was observed that PM9 was the only treatment that significantly reduced incidence of damping off and anthracnose mixed diseases on pepper and improved root colonization [24]. The consortium of rhizobacteria exhibited distinct differences in their efficacy for controlling damping off and anthracnose (Table 1). Before seven days of harvesting the crop, incidence and severity of damping off in uninoculated control plots were 7.06% and 8.75%, respectively, while those of anthracnose ranged from 8.01% and 9.70%. However, plots that received bio-inoculation of *Bacillus pumilus* and *Bacillus subtilis* had incidence and severity of 1.81% and 0.62% for damping off and 1.75% and 0.69% for anthracnose. Bio-inoculated plots also observed reduced incidence and severity by 94.50% (damping off) and 95.64% (anthracnose). This suggested that the consortium exhibited better disease control efficacy over the commercially available fungicides (78.80–79.26% for damping off and 80.32–82.65% for anthracnose), fumigation (77.35% for damping off and 81.80% for anthracnose) and soil solarization (74.56% for damping off and 84.40% for anthracnose) practices utilized for disease control. It is because of the antagonistic microbes (*Bacillus pumilus* and *Bacillus subtilis*) in the rhizosphere which protect the host plant by directly suppressing the growth and proliferation of phytopathogens [25,26]. Competitive root tip colonization by rhizobacterial strains might play a crucial role in the efficient control of soil-borne diseases [27]. Under field conditions, it was also investigated whether *Bacillus* isolates have a potential to be used as biobacteriocides [28] and for the control of red rot [29]. Principal coordinate analysis (PCA) was used to investigate the relationships between applied treatments with growth and yield of bell pepper as well as disease suppression of damping off and anthracnose.

**Figure 6:**
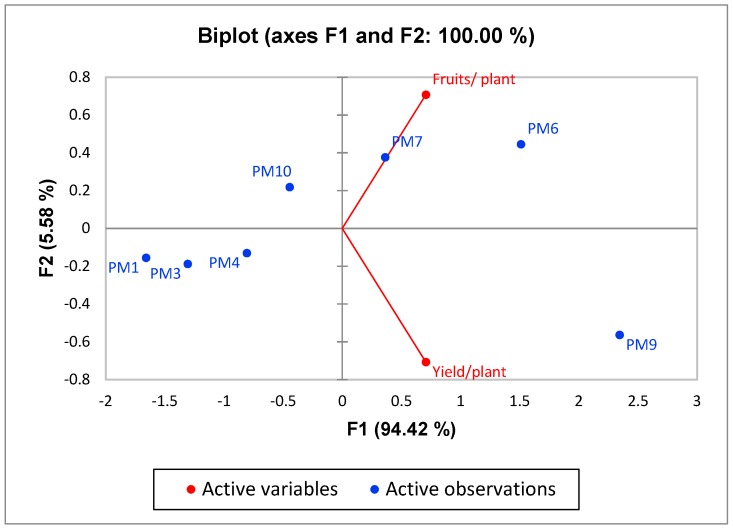
Principal component analysis (PCA) of growth and yield characteristics of capsicum. PM1—Control, PM3—Benomyl, PM4—Captan, PM6—Recommended dose of NPK, PM7—Soil fumigation with MeBr (425 kg/ha broadcast 98:2 MeBr:chloropicrin), PM9—PGPR’s consortium (*Bacillus pumilus* and *Bacillus subtilis*), PM10—Soil solarization.

**Figure 7:**
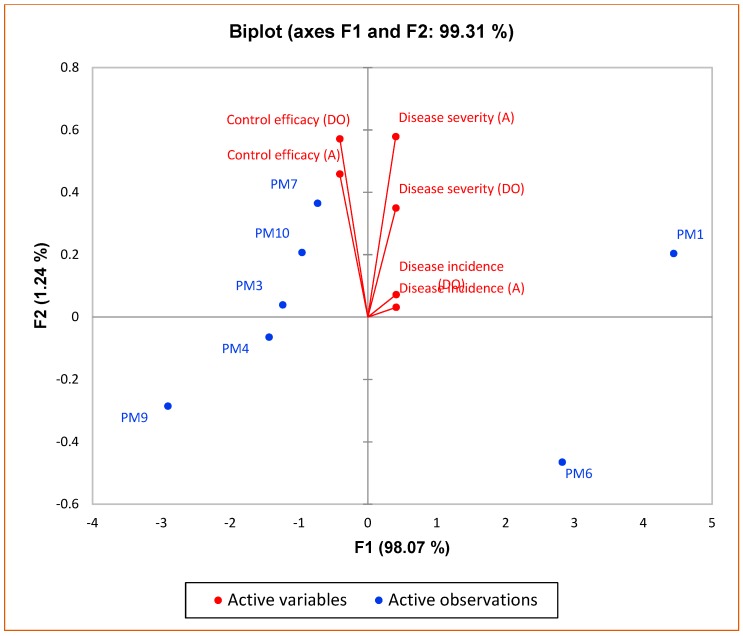
Principal component analysis (PCA) for control of damping off and anthracnose. DO—damping off, A—anthracnose.

PCA1 of growth and yield parameters of bell pepper revealed that principal component (PC1) and principal component (PC2) accounted for 94.42% and 5.58% of the data variation, respectively (Figure 6). PC1 comprised treatments with PM6 and PM7, and it showed a strong relation with fruit yield/plants. In PC2, PGPR’s consortium (*Bacillus pumilus* and *Bacillus subtilis*) showed more influence on yield/plant. PCA2 of disease suppression of damping off and anthracnose in bell pepper revealed that principal component (PC1) and principal component (PC2) accounted for 98.07 and 1.24% of the data variation, respectively (Figure 7). PC1 allowed the comparison of two treatments: Recommended dose of fertilizers and no fertilizers. Both treatments showed more influence on diseases severity and incidence for damping off and anthracnose. PC2 showed that PGPR’s consortium (*Bacillus pumilus* and *Bacillus subtilis*) had a significant influence on control efficacy of both damping off and anthracnose diseases. High impact of disease incidence and severity were seen in PC2 for both damping off and anthracnose. The second component was related to the very low disease control efficacy exhibited by control and recommended dose of fertilizers. This analysis showed that inoculation with PGPR’s consortium (*Bacillus pumilus* and *Bacillus subtilis*) had significant effect not only on growth and yield enhancement of bell pepper but also against the diseases (damping off and anthracnose diseases) caused by soil-borne fungal pathogens.

## 4. Conclusions

This study underlines the importance of PGPR’s consortium (*Bacillus pumilus* and *Bacillus subtilis*) for multiple PGP (plant growth promoting) and biocontrol traits and evaluates their potential through field experiments in the bell pepper. In this study, based on in vitro and field experiments, PGPR’s consortium (*Bacillus pumilus* and *Bacillus subtilis*) markedly enhanced growth, suppressed damping off and anthracnose disease incidence, and increased fruit yield in bell pepper compared to other fertilizers and fungicidal treatments. Therefore, it is apparent that PGPR’s consortium (*Bacillus pumilus* and *Bacillus subtilis*) receives strong assurance as a viable substitute to chemical fertilizers and can be integrated into pertinent nutrient and disease management schedules for bell pepper.

## Figures and Tables

**Table 1:** Efficacy of PGPR’s consortium to control damping off and anthracnose in capsicum under field conditions.

Treatments	Damping Off			Anthracnose		
	Disease Incidence (%)	Disease Severity (%)	Control Efficacy	Disease Incidence (%)	Disease Severity (%)	Control Efficacy
PM1	7.06 ± 0.38	8.75 ± 0.24	24.83 ± 1.89	8.01 ± 0.41	9.70 ± 0.26	22.75 ± 2.01
PM3	2.96 ± 0.21	3.47 ± 0.41	78.80 ± 0.46	2.70 ± 0.23	2.41 ± 0.24	80.32 ± 1.25
PM4	2.86 ± 0.17	3.10 ± 0.03	79.26 ± 0.38	2.79 ± 0.36	1.78 ± 0.28	82.65 ± 2.46
PM6	5.80 ± 0.34	6.44 ± 0.21	29.60 ± 1.05	6.10 ± 0.37	7.20 ± 0.22	31.29 ± 1.21
PM7	3.44 ± 0.31	3.96 ± 0.42	77.35 ± 2.51	3.22 ± 0.20	3.90 ± 0.25	81.80 ± 3.48
PM9	1.81 ± 0.31	0.62 ± 0.01	94.50 ± 1.08	1.75 ± 0.29	0.69 ± 0.10	95.64 ± 1.07
PM10	2.98 ± 0.19	3.55 ± 0.37	74.56 ± 1.63	3.29 ± 0.67	3.41 ± 0.29	84.40 ± 2.44
LSD	0.905	0.702	7.532	0.916	0.605	8.364

PM1—Control, PM3—Benomyl, PM4—Captan, PM6—Recommended dose of NPK, PM7—Soil fumigation with MeBr (425 kg/ha broadcast 98:2 MeBr:chloropicrin), PM9—PGPR’s consortium (*Bacillus pumilus* and *Bacillus subtilis*), PM10—Soil solarization
